# An Enhanced Lateral Flow Assay Based on Aptamer–Magnetic Separation and Multifold AuNPs for Ultrasensitive Detection of *Salmonella* Typhimurium in Milk

**DOI:** 10.3390/foods10071605

**Published:** 2021-07-11

**Authors:** Pingping Gao, Lihan Wang, Yang He, Yitian Wang, Xinyan Yang, Shiqian Fu, Xue Qin, Qing Chen, Chaoxin Man, Yujun Jiang

**Affiliations:** Key Laboratory of Dairy Science, Ministry of Education, Department of Food Science, Northeast Agricultural University, Harbin 150030, China; 18374844214@168.com (P.G.); wang_lihan@163.com (L.W.); hy0130a@163.com (Y.H.); omnsci_w@163.com (Y.W.); 15545116996@163.com (X.Y.); shiqian_fu@163.com (S.F.); qinxue1996316@163.com (X.Q.); chenqingchen163@163.com (Q.C.); cxman@neau.edu.cn (C.M.)

**Keywords:** enhanced lateral flow assay, multifold AuNPs, magnetic separation, *S.* Typhimurium

## Abstract

In this paper, a novel and ultrasensitive lateral flow assay (LFA) based on aptamer–magnetic separation, and multifold Au nanoparticles (AuNPs) was developed for visual detecting *Salmonella enterica ser.* Typhimurium (*S.* Typhimurium). The method realized magnetic enrichment and signal transduction via magnetic separation and achieved signal amplification through hybridizing AuNPs–capture probes and AuNPs–amplification probes to form multifold AuNPs. Two different thiolated single-strand DNA (ssDNA) on the AuNPs–capture probe played different roles. One was combined with the AuNPs–amplification probe on the conjugate pad to achieve enhanced signals. The other was connected to transduction ssDNA1 released by aptamer–magnetic capture of *S.* Typhimurium, and captured by the T-line, forming a positive signal. This method had an excellent linear relationship ranging from 8.6 × 10^2^ CFU/mL to 8.6 × 10^7^ CFU/mL with the limit of detection (LOD) as low as 8.6 × 10^0^ CFU/mL in pure culture. In actual samples, the visual LOD was 4.1 × 10^2^ CFU/mL, which did not carry out nucleic acid amplification and pre-enrichment, increasing three orders of magnitudes than unenhanced assays with single–dose AuNPs and no magnetic separation. Furthermore, the system showed high specificity, having no reaction with other nontarget strains. This visual signal amplificated system would be a potential platform for ultrasensitive monitoring *S.* Typhimurium in milk samples.

## 1. Introduction

*Salmonella* contamination is a significant public health problem globally. It is spread primarily through dairy products and foods of animal origin and can cause gastroenteritis, typhoid fever, and even mortality in humans and animals [1,2]. *Salmonella* spp. outbreaks are estimated to be associated with about 85% (80.3 million cases) of foodborne diseases (93.8 million cases) worldwide each year, resulting in over 100,000 deaths and incurring a considerable economic cost to society [3]. Of more than 2500 serotypes of salmonella, almost all are pathogenic, but their hosts are usually different [4,5]. *S.* Choleraesuis and *S.* Dublin have a single host, such as pigs and cattle; *S. Enteritidis* and *S.* Typhimurium are the most aggressive bacteria that can spread from animals to humans, causing a wide range of diseases [6]. Moreover, *S.* Typhimurium outbreaks after consuming tainted milk are rather common [7]. *S.* Typhimurium contaminated milk has caused over 100 outbreaks of foodborne illness in China over the past decade, resulting in 750 cases and 215 hospitalizations in China [1,8]. Therefore, it is necessary to establish a simple, rapid, and sensitive method for the detection of *S.* Typhimurium in milk.

Lateral flow assay (LFA) has drawn wider attention in foodborne pathogen detection [9,10] for its rapidity, convenience, lower-cost efficiency, and easy interpretation of its results [2,11,12]. Au nanoparticles (AuNPs) have been the most commonly used marker in LFA due to their good biocompatibility and high molar absorption coefficient. However, due to the weak optical signal intensity and poor matrix tolerance of AuNPs, LFA is challenging to meet the criteria in trace quantities in real sample detection [13]. Fluorescent materials such as quantum dots (QDs) [14] and upconverting nanoparticles (UCNPs) [15] have high sensitivity, but their uses have been hampered by chemical instability, large reader required, and the complexity of coupling recognition molecules. Hence, AuNPs still occupy the irreplaceable status due to their visibility, nontoxicity, and simple marking. Although certain research studies on AuNPs-based signal amplification, such as gold growth [16] and gold polymerization [17], have improved sensitivity, they have flaws in controllability, reproducibility, stability, and simplicity. Gold conjugates [9,18] depending on the coupling between antibodies; there are also defects in their stability and operation. As far as we know, the research on the aggregation of AuNPs based on the hybridization of functional nucleic acid strands has been rare.

Immunomagnetic separation (IMS) is commonly used as a sample pretreatment step prior to LFA detection, and it has proven to be an effective strategy for reducing the matrix effect using an external magnetic field [19,20,21]. In IMS, antibodies are usually used for identification, and the isolated target bacteria be used for nucleic acid amplification or direct detection, which has the disadvantages of poor stability, complex operation, and color blocking of macromolecules [22,23]. The selected aptamers with high affinity and specificity in vitro could be resynthesized with no difference across batches for further research. As a result, they are growing popular as antibody alternatives [4,5,24].

The objective of this research is to improve the sensitivity of the test strip and simplify the detection. An enhanced signal was formed by the hybridization of two kinds of AuNPs–probes; meanwhile, the use of aptamer–magnetic separation minimized the matrix effect. The scheme in this work is straightforward, controllable, efficient, visible, and with low requirements, thus showing great potential in the application for detecting *S.* Typhimurium in milk on-site.

## 2. Materials and Methods

### 2.1. Chemicals and Materials

Streptavidin magnetic nanoparticles (SMNPs) (1.0 μm in diameter, 10 mg/mL) were obtained from Invitrogen Biotechnology Co., Ltd. (Carlsbad, CA, USA). Salmon sperm DNA, tRNA, and Tris were purchased from Solarbio Science & Technology Co., Ltd. (Beijing, China). AuNPs (15 nm) were acquired from Jieyi Biotechnology Co., Ltd. (Shanghai, China). Ethylene diamine tetraacetic acid (EDTA) was received from Saiguo Biotech Co., Ltd. (Guangzhou, China). The sample pad, conjugate pad, nitrocellulose membrane, absorption pad, and polyvinyl chloride pad were provided from Hangzhou Goodhere Biotechnology Co., Ltd. (Hangzhou, China). Aptamers and ssDNA were synthesized by Sangon Biotech Co., Ltd., (Shanghai, China); sequences are shown in Table 1. Polyvinylpyrrolidone (PVP) and sodium dodecyl sulfate (SDS) were also obtained from Sangon Biotech Co., Ltd. Tween-20, D-trehalose, Tris-HCl, and sucrose were acquired from Yihui Biological Technology Co., Ltd. (Shanghai, China). Trisodium citrate was purchased from Sinopharm Chemical Reagent Company (Beijing, China). Streptavidin was acquired from Sigma Co., Ltd (Silicon Valley, CA, USA). Disodium phosphate, sodium dihydrogenphosphate was obtained by Tianli Chemical Reagent Co., Ltd. (Tianjin, China). All chemicals used in this study were of analytical reagent grade.

### 2.2. Equipment

Spray test (T) and control (C) lines using the XYZ 3050 strip dispenser produced by BioDot (Irvine, CA, USA). Use the HGS201 programmable slitting machine purchased from Autokun (Los Angeles, CA, USA) to cut the strips. The T/ C-line signal was measured using a chromatographic instrument acquired by Weifang Bainuo Di Biotechnology Co. Ltd. (Weifang, China). Using HT7700 Exalens Transmission electron microscope (TEM) (Hitachi, Tokyo, Japan) to view the shape of original AuNPs and AuNPs–probe. A high-speed freezing centrifuge was provided from Sigma Laborzentrifugen Co., Ltd. (Osterode am Harz Niedersachsen, Germany). Desktop full temperature shaking incubator was provided from Labotery Instrument Equipment Co., Ltd., (Tianjin, China). Infinite 200 PRO multifunctional microplate reader was obtained from Tecan Trading AG, (Switzerland, Europe).

### 2.3. Bacterial Cultures

*S.* Typhimurium ATCC 14028 (ATCC, American Type Culture Collection) was used as target strain in this study. A single colony of strains from Luria Bertani (LB) agar (Haibo Biological Technology Co., Ltd., Qingdao, China) was cultured in LB Broth in Erlenmeyer flask overnight at 37 °C with Desktop full temperature shaking incubator at 150 rpm before use. Strains cultured in LB medium were centrifuged (8000× *g*, 4 °C, 5 min)and resuspended in PBS buffer. The number of viable bacteria was measured by plating count on LB agar at 37 °C.

### 2.4. Preparation of SMNPs–Aptamer–ssDNA1

8 μM aptamer and 8 μM ssDNA1 were mixed in 20 μL Tris-HCl (100 Mm, pH 7.4), then the mixture incubated with 150 rpm at 25 °C for 2 h to form the aptamer–ssDNA1 complex. A 25 μL of SMNPs was added into silicide tube and washed three times with 1× B&W buffer (5 mM Tris-HCl, 0.5 mM EDTA, 1 M NaCl, pH 7.5), then fixed to 50 μL with 2× B&W buffer (10 mM Tris-HCl, 1 mM EDTA, 2 M NaCl, pH 7.5). Added aptamer–ssDNA1 complex prepared above, reacting for 15 min at 25 °C with 250 rpm to make up SMNPs–aptamer–ssDNA1. The SMNPs–aptamer–ssDNA1 reacted with the blocking agent (Salmon Sperm DNA (1 mg/mL) and yeast tRNA (0.1 mg/mL), (pH 9) for 30 min to reduce the nonspecific binding. Under the action of an external magnetic field, the supernatant was removed. The precipitate was resuspended to 50 μL with PBS and then stored at 4 °C for further use.

### 2.5. Magnetic Enrichment of S. Typhimurium

Centrifuged (8000× *g*, 4 °C, 5 min) *S.* Typhimurium cultured to 10^6^ CFU/mL to remove the supernatant, after washing with PBS buffer, mixed with SMNPs–aptamer–ssDNA1 and incubated at 37 °C for 90 min. Then, the mixture was placed on the magnetic frame, and the supernatant containing ssDNA1 was drawn for the next experiment. The precipitate was fixed with PBS to 1 mL for plates counting and calculating the capture rate. The capture rate was calculated using the following formula:

Capture rate (%) = W_0_/W × 100%

where W_0_ (CFU/mL) represented the number of viable cells separated by magnetic separation, and W (CFU/mL) was the number of original viable cells.

### 2.6. Preparation of AuNPs–Probe

Based on previous research [25], a modified slightly preparation method of AuNPs–probe was used. First, 8 μL thiolated ssDNA2 (100 μM) was mixed with 20 mM Tris (2-carboxyethyl) phosphine (TCEP) (10 μL) to activate the thiol group. Then, 1 mL AuNPs was added and reacted at 50 °C for 22 h to synthesize the amplification probe. A 100 μL 0.1 M phosphate buffer A (containing 0.1% (*m*/*v*) SDS, pH 7.6) was provided for 1 h, and then the 0.01 M phosphate buffer B (containing 0.01% SDS, 1 M NaCl) was added in portions until the concentration of NaCl was 0.06 M to age. The mixture was centrifuged (12,000× *g*, 4 °C, 30 min) to remove the supernatant, and washed with 500 μL purified water. After centrifuging (12,000× *g*, 4 °C, 20 min) again to remove excess ssDNA2, the pellet was dissolved in the 0.05 M phosphate buffer C (containing 0.05% (*m*/*v*) SDS, pH 7.6), stored in the dark at 4 °C for further use. Identically, 8 μL thiolated ssDNA3 (100 μM) and ssDNA4 (100 μM) were processed in the same way to synthesize the capture probe. The synthesis of the amplification probe and capture probe was characterized by the microplate reader and TEM.

### 2.7. Assembly Lateral Flow Assay Test Strips

**Figure 1:**
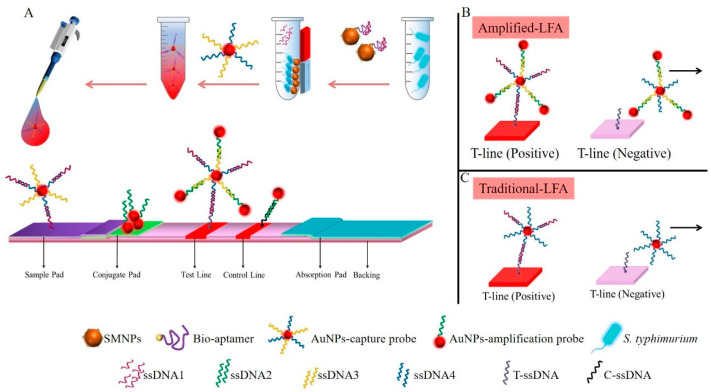
Lateral flow assay enhanced by multifold AuNPs combined with magnetic enrichment for *Salmonella* Typhimurium detection. Schematic illustration of the detection of *Salmonella* Typhimurium using AuNPs aggregates enhanced lateral flow assay (**A**); multifold AuNPs on test line in the presence (**left**) and absence (**right**) of *Salmonella* Typhimurium (**B**); single–dose AuNPs on the test line in the unenhanced lateral flow assay (**C**).

As shown in Figure 1A, the LFA test strips for *S.* Typhimurium were composed of a sample pad, conjugate pad, nitrocellulose (NC) membrane, and absorbent pad, where the two ends of each part overlapped by about 3 mm. In order to prepare the LFA strips, the sample pad and conjugate pad were soaked in the 100 mM Tris buffer (containing 2% (*m*/*v*) D-trehalose, 2% (*m*/*v*) sucrose, 1% (*m*/*v*) PVP, 0.5% (*v*/*v*) Tween-20, 0.2% (*m*/*v*) BSA, pH 8) for 1 h, and dried at 37 °C. Afterward, a conjugate pad was dipped in the amplification probe solution and then dried for 45 min at 37 °C. Next, 50 μL T-ssDNA (10 μM) and C-ssDNA (10 μM), respectively, were mixed with 10 μL streptavidin (1 mg/mL) and then incubated at 37 °C for 2 h. The solution was finally distributed onto an NC membrane to form a test line (T-line) and a control line (C-line), and the distance between the two lines was 8 mm. Then, the prepared master cards were cut into separate strips (length 5 cm, width ∼ 0.4 cm) by a paper slitting machine. Strips were then stored at 4 °C in a sealed plastic bag with desiccant gel for further use.

### 2.8. Detection of S. Typhimurium in Pure Culture

#### 2.8.1. Detection Sensitivity

Under optimal conditions, detections of *S.* Typhimurium in PBS buffer were carried out. *S.* Typhimurium was diluted to 8.6 × 10^7^–8.6 × 10^−1^ CFU/mL. The supernatant of 12 μL SMNPs–aptamer–ssDNA1 was removed and incubated with 100 μL *S.* Typhimurium at 37 °C for 90 min. Then, they were put on a magnetic frame for 2 min, and the supernatant was mixed with the capture probe for 15 min at 37 °C. The above compound was transferred to LFA for further detection. After 15 min, the LFA test trip was inserted into a chromatographic instrument to analyze the recording of the Molar absorbance (mABS) of the red band on the T-line and quantify the strains. The control groups were PBS. Each experiment was repeated three times.

Unenhanced LFA was also analyzed as a comparison. Aptamer–ssDNA1 was incubated with the *S.* Typhimurium for 90 min and then centrifuged (8000× *g*, 4 °C, 10 min) to obtain the supernatant. The capture probe linking no ssDNA3 reacted with the supernatant at 37 °C for 30 min. The rest operation was consistent with the enhanced LFA. The control groups were PBS. Each experiment was repeated three times.

#### 2.8.2. Detection Specificity

In total, 13 common microorganisms were selected to evaluate the specificity of the method, including *Salmonella* Typhimurium CMCC 50222 (CMCC, China Medical Culture Collection (Beijing, China)), *Salmonella* Enteritidis CICC 21482 (CICC, China Center of Industrial Culture Collection), *Salmonella* Dublin CMCC 50042, *Salmonella* Dublin CMCC 50092, *Escherichia coli* O157:H7 ATCC 25922, *Bacillus cereus* CMCC 63303, *Enterobacter aerogenes* ATCC 13048, *Cronobacter sakazakii* ATCC 29544, *Listeria monocytogenes* ATCC 19112; *Listeria welshimeri* ATCC 43550; *Staphylococcus aureus* ATCC 13565; *Shigella flexneri* CMCC 51572; *Vibrio parahaemolyticus* ATCC 17802. All strains were cultured to 10^6^ CFU/mL in LB medium and analyzed by the above methods. All experiments were performed three times.

### 2.9. Detection of S. Typhimurium in Milk

Ultra-high temperature sterilized (UHT) milk bought in the local supermarket (Harbin, China) was tested for *S.* Typhimurium by conventional culture methods (ISO 6579:2017). Briefly, 1 mL different concentrations of *S.* Typhimurium were inoculated into 24 mL sterile milk, making the final concentration 4.1 × 10^7^–4.1 × 10^1^ CFU/mL, whereas the control group was not spiked with *S.* Typhimurium. The 1 mL spiked samples were centrifuged (8000× *g*, 4 °C, 5 min) to remove fat and resuspended with PBS to obtain experimental milk samples. The obtained milk samples were detected by enhanced LFA and unenhanced LFA, respectively. All experiments were performed three times.

### 2.10. Statistical Analysis

All the measurements were performed in triplicate and expressed as mean ± standard deviation (SD). Statistical analysis was performed using SPSS statistical 20.0 software (SPSS Inc., Chicago, IL, USA). Statistical significance was defined at *p* < 0.05.

## 3. Results

### 3.1. The Principle of Enhanced Lateral Flow Assay

By coupling aptamer–magnetic separation with multifold AuNPs, researchers were able to develop enhanced LFA. SMNPs–aptamer–ssDNA1 was prepared to complete the enrichment and signal transduction of *S.* Typhimurium. Among them, ssDNA1 was the based pair to the aptamer and opened the secondary structure of the aptamer. When *S.* Typhimurium was present, and the aptamer developed a secondary structure and formed a specific bond with the strain, releasing ssDNA1 [24]. It is well known that the generation of detectable signal output in the T-line depends on the sufficient accumulation of AuNPs. The number of accumulated AuNPs reaches the colorimetric threshold and can be easily identified by naked eyes [26]. As a result, it is necessary to increase the accumulation of AuNPs in order to further amplify the colorimetric signal intensity of AuNPs to produce detectable red bands caused by unit analytes. Therefore, researchers prepared two kinds of AuNPs–probes complementary to each other to increase the aggregation of AuNPs. First, the amplification probe made of AuNPs and one kind of ssDNA (ssDNA2) performed the function of signal amplification. In the second approach, two kinds of ssDNA were functionalized to the surfaces of AuNPs to form a capture probe, which included a detection ssDNA (ssDNA4) partially complementary to target ssDNA1 and an auxiliary ssDNA (ssDNA3) for gathering amplification probe.

Figure 1A schematically illustrates the process of the aptamer–magnetic separation with multifold AuNPs based LFA. A sandwich immunoassay format was used for the assay. When the *S.* Typhimurium was present, the SMNPs–aptamer efficiently enriched them, releasing the transduction ssDNA1. Subsequently, the end of ssDNA1 was connected to the capture probe through base pairing with ssDNA4. Then, the complex was placed on the sample pad and migrated through capillary force. When flowed through the conjugate pad, the amplification probe linked to ssDNA3 to form multifold AuNPs and then continued to migrate. The free end of ssDNA1 was captured by T-ssDNA, forming a bright red band on the T-line. The excessive amplification probe on conjugate pad chromatograph to the C-line was captured by C–ssDNA and displayed as a red band, which prove the effectiveness of the detection. In the absence of *S.* Typhimurium, the multifold AuNPs would flow past the T-line without interacting, and the amplification probe would be captured by the C–ssDNA (Figure 1B). However, the unenhanced LFA has only single-dose AuNPs at the T-line, as shown in Figure 1C.

### 3.2. Optimization of Capture Conditions

**Figure 2:**
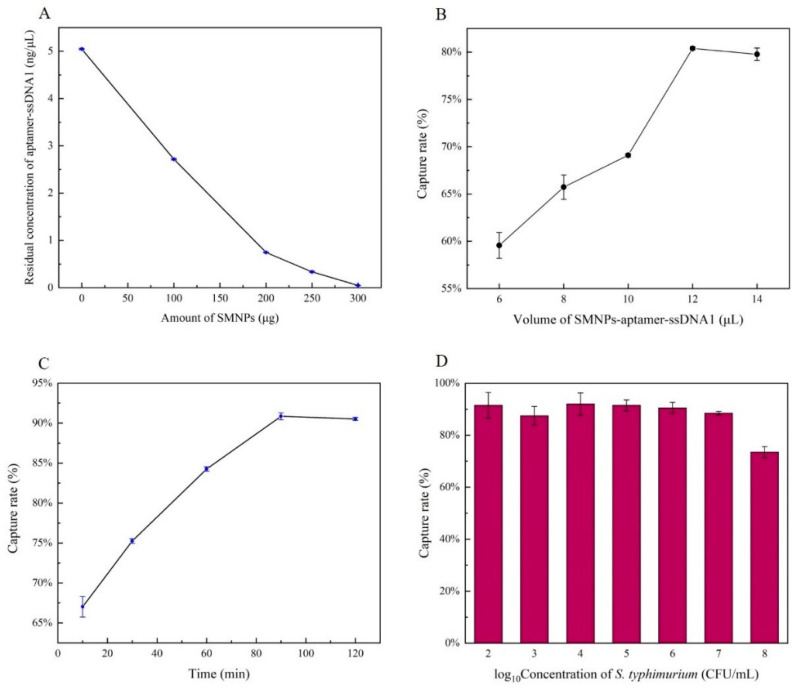
Optimization of capture rate for SMNPs–aptamer–ssDNA1 against *Salmonella* Typhimurium. Effect of the amount of SMNPs on residual aptamer–ssDNA1 concentration (**A**); effect of the volume of SMNPs–aptamer–ssDNA1 on capture rate (**B**); effect of the reaction time on capture rate (**C**); effect of the concentration of *S.* Typhimurium on capture rate (**D**); data represent mean ± SD (*n* = 3). Error bars indicate the standard errors of three independent experiments.

The amount of SMNPs was optimized to allow the aptamer–ssDNA1 generated in advance to be completely integrated. As shown in Figure 2A, with the increase of SMNPs to 250 μg, the concentration of aptamer–ssDNA1 in supernatant decreased significantly to 0.07 ng/μL; then, the amount of residual SMNPs in the solution was too small to be connected. Therefore, 250 μg SMNPs were selected as the optimum.

The concentration of ssDNA1 released in the supernatant increased with the increase of capturing bacteria rate. Therefore, the volume of SMNPs–aptamer–ssDNA1 and capture time were optimized to obtain the optimum efficiency.

As the volume of SMNPs–aptamer–ssDNA1 increased to 12 μL, the capture rate was significantly improved (Figure 2B). When the volume continued to increase, the capture rate stabilized, indicating that 12 μL was the optimal capture volume. Then, we evaluated the effect of magnetic capture time on capture rate, as shown in Figure 2C. With the passage of time, the capture efficiency kept increasing, reaching the threshold at 90 min. In this case, the capture efficiency was 92%. Figure 2D showed the capture efficiency of 12 μL SMNPs–aptamer–ssDNA1 at the different concentrations of *S.* Typhimurium. The capture efficiency of all concentrations except 10^8^ CFU/mL was above 90%, which indicated the feasibility of this method. The reason for the low capture rate of 10^8^ CFU/mL was that the SMNPs–aptamer–ssDNA1 had reached saturation and could not isolate the excess bacteria.

### 3.3. Characterization of the AuNPs–Probe

**Figure 3:**
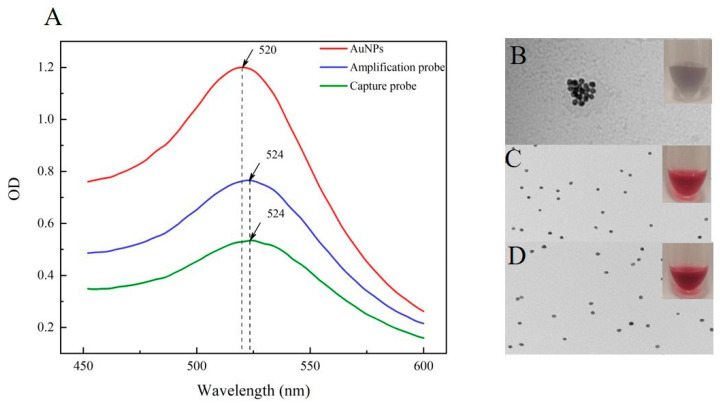
Characterization of the AuNPs and AuNPs–probe: UV absorption spectra peak alteration of original AuNPs, amplification probe and capture probe (**A**); TEM images and photographs of original AuNPs (**B**), amplification probe (**C**), and capture probe (**D**) reaction with 2 M NaCl.

The two prepared AuNPs–probe and original AuNPs solutions were characterized by TEM and microplate reader. In this research, the 5’-end of ssDNA2, ssDNA3, and ssDNA4 were modified with a sulfhydryl, which could be covalently attached to the AuNPs through Au–S bonds. The surface of AuNPs was covered with citric acid molecules. When the connected ssDNA was insufficient, there would still be negative charge exposure, and the presence of Na^+^ would occur aggregation, the solution would appear purple [27]. Conversely, if sufficient ssDNA was coated on the surface of AuNPs, the color would remain stable bright red, antagonizing salt in the solution. After 2 M NaCl was added, the AuNPs solution appeared purple gray, with black particles occurring immediately, and clear particle aggregation could be seen through TEM (Figure 3B). However, the capture probe and amplification probe remained transparent and bright red, with no aggregation, as mentioned before, and good dispersion under TEM (Figure 3C,D); this was the same as the result of the previous study [28]. For further quantification, the results of UV analysis were shown in Figure 3A. Compared to the original AuNPs, the absorption peak of the two probes was significantly red-shifted from 520 nm to 524 nm, which indicated the successful synthesis of the capture probe and the amplification probe.

### 3.4. Optimization of Analysis Parameters for LFA

The hybridization of two AuNPs probes, which played a crucial role in signal amplification, was used to achieve AuNP aggregation in this study. Therefore, the proportion between ssDNA3 and ssDNA4 on the capture probe and the concentration of the amplified probe were optimized to maximize the sensitivity. The analytical performance was evaluated by the mABS of the T-line and C-line of the LFA.

In this work, ssDNA3 and ssDNA4 linked to AuNPs played different roles. The ratio between them determined the detection performance of the LFA. There was a competitive relationship between ssDNA3 and ssDNA4. ssDNA3 can be combined with the amplification probe on the conjugate pad to amplify the signal, and the increased amount of it will promote the binding of more amplification probes. Although insufficient ssDNA3 improves the capacity to capture the target, it will cause the aggregation rate of AuNPs in the T-line to decrease [29]. Excessive ssDNA3 would result in a ssDNA4 deficiency, which would lead to a ssDNA1 shortage and finally cause the attenuated signal of the T-line. Moreover, excessive accumulation of AuNPs would lead to increased migration resistance, which would indirectly reduce the T-line response value [30].

**Figure 4:**
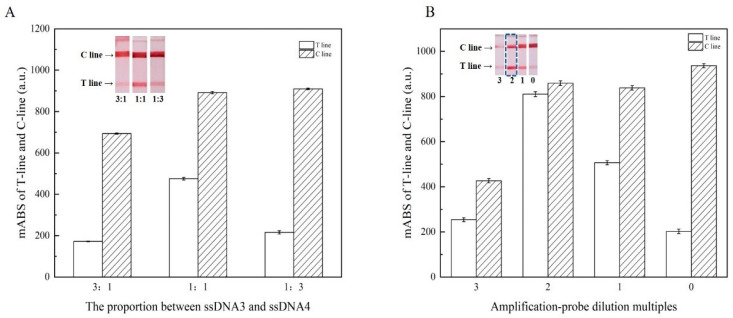
Optimization of the analysis parameters: Effect of proportion between ssDNA3 and ssDNA4 of amplification probe on the signal of lateral flow strips (**A**); effect of amplification probe dilution multiples on the signal of lateral flow strips (**B**). Inset: photographs of the lateral flow assay results of different parameters. Data represent mean ± SD (*n* = 3). Error bars indicate the standard errors of three independent experiments.

Through the consequence of three molar ratios, the maximum response value of the T-line was obtained, as shown in Figure 4A. With the increase of ssDNA3, the response value of the T-line first increased and then decreased, and the value reached the maximum at 1:1. When ssDNA3 continued to increase, the signal of the T-line diminished. 1:1 was chosen as the most suitable proportion. 

Amplification probes played a vital role in this assay, which affected amplification efficiency and resulted in sensitivity variations. In order to obtain high sensitivity while avoiding excessive amounts of AuNPs, four kinds of dilution multiples of amplification solutions were explored in this study. As can be seen in Figure 4B, the highest signal of the T-line was at 2 multiples, while the T-line and C-line both showed bright red, but the obvious reduction in the T-line was observed in less than 2 multiples. When the amplification probe was in high concentration, it was easier to accumulate when drying, which led to the increase in resistance in the reaction process, and therefore, it cannot be better released. The fluidity of the multifold AuNPs generated on the conjugate pad was worse, which weakened the color of the T-line; at the low concentration, it would not combine with the capture probe completely, resulting in insufficient color.

### 3.5. Sensitivity of the Lateral Flow Assay

**Figure 5:**
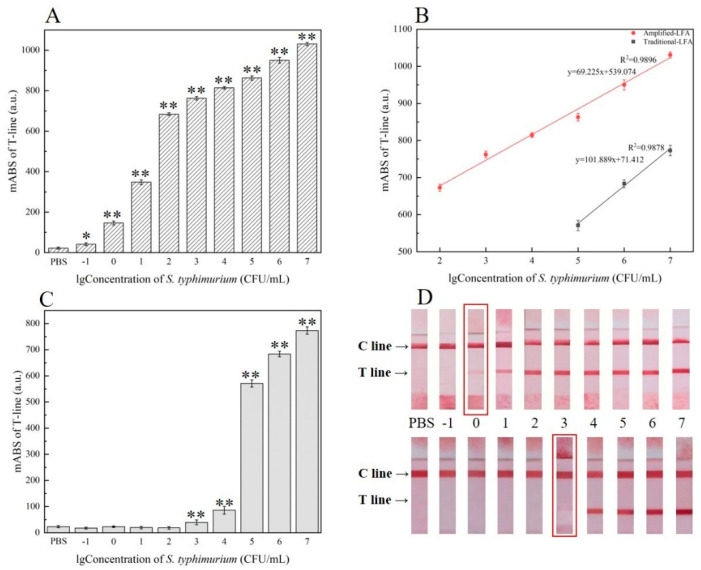
Analytical characteristics of the enhanced lateral flow assay and unenhanced lateral flow assay: Sensitivity of the enhanced lateral flow assay (**A**) and unenhanced lateral flow assay (**C**) when different *S.* Typhimurium concentration was applied; data represent mean ± SD (*n* = 3, * *p* < 0.05, ** *p* < 0.01 vs. PBS group). Linear response of the two kinds of strips (enhanced and unenhanced) for *S.* Typhimurium detection (**B**); photographs of the enhanced detection (up) and unenhanced detection (down) for 8.6 × 10^−1^–8.6 × 10^7^ CFU/mL *S.* Typhimurium in PBS (**D**). Data represent mean ± SD (*n* = 3). Error bars indicate the standard errors of three independent experiments.

Different concentrations (from 10^7^–10^−1^ CFU/mL) of *S.* Typhimurium were detected by the enhanced LFA under optimal conditions to determine the sensitivity of the system. The response value of the T-line grew progressively as the concentration of *S.* Typhimurium increased, as expected. We can infer from Figure 5B that in the concentration range of 8.6 × 10^2^–8.6 × 10^7^ CFU/mL, the calibration curve showed that the response value of the T-line had a great linear relationship with the logarithmic value of the target concentration and a better correlation coefficient (*R*^2^ = 0.9896), y = 69.225x + 539.074 (*n* = 3), which allowed qualitative and quantitative detection of *S.* Typhimurium. The limit of detection (LOD) was proved to be 8.6 × 10^−1^ CFU/mL (Figure 5A), while the visual detection limit was 8.6 × 10^0^ CFU/mL (Figure 5D). Clearly, the curve of conventional analysis lay below the amplification curve, the detection ranged from 8.6 × 10^5^ to 8.6 × 10^7^ CFU/mL (Figure 5B), with the LOD 8.6 × 10^3^ CFU/mL (Figure 5C,D), which implies that the sensitivity was lower than the novel assay with a wider linear detection range. In sensitivity verification, although the ssDNA3 were added to capture probe of amplified LFA, which may have caused some targets to escape at high concentrations. However, the amplified signal was significantly bigger than the unamplified signal at high concentrations of the target bacteria, indicating that ssDNA3 improved the AuNPs trapping ability of the capture probe. While the target ssDNA1 could be fully captured at the low concentration, the signal intensity was still higher than that of the enhanced LFA, which further proved that the multifold AuNPs could obtain higher sensitivity [31]. Moreover, the introduction of magnetic separation greatly reduced matrix interference and efficiently enriched *S.* Typhimurium. Therefore, the improved LFA could be particularly useful for high sensitivity on-site detection of *S.* Typhimurium, especially in remote environments.

### 3.6. Specificity of the Enhanced LFA

In order to verify the specificity of this method, 2 strains of *S.* Typhimurium and 12 strains of other common foodborne pathogens were tested. As shown in Table 2, all strains were negative except for *S.* Typhimurium, and no cross reaction occurred even at high concentrations, which showed that our system was highly specific to *S.* Typhimurium. This high specificity was closely related to the high selectivity of the aptamer.

### 3.7. Detection in Milk Samples

**Figure 6:**
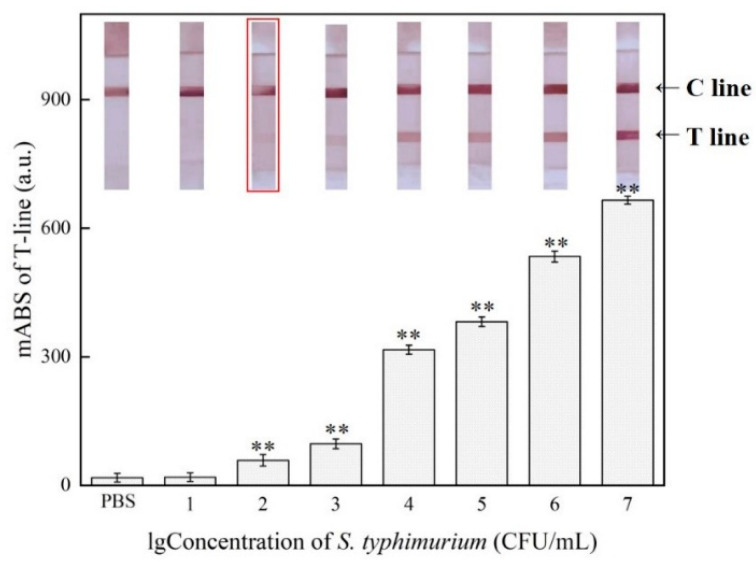
Sensitivity of the enhanced lateral flow assay against *S.* Typhimurium at 4.1 × 10^1^–4.1 × 10^7^ CFU/mL in milk. Inset: photographs of the lateral flow assay results of *S.* Typhimurium at 4.1 × 10^1^–4.1 × 10^7^ CFU/mL in milk; data represent mean ± SD (*n* = 3, ** *p* < 0.01 vs. PBS group). Error bars indicate the standard errors of three independent experiments.

We further evaluated the application of the proposed method in actual milk samples. The concentration of *S.* Typhimurium in the milk sample was changed from 4.1 × 10^1^ to 4.1 × 10^7^ CFU/mL. As shown in Figure 6, when the concentration of *S.* Typhimurium reached 4.1 × 10^2^ CFU/mL, the response of the T-line was higher than that of the negative control by the reader, and the T-line began to turn red, and the color deepened as the concentration increased, while the LOD of unenhanced LFA was only 4.1 × 10^5^ CFU/mL. The results suggested that it was possible for our proposal to detect *S.* Typhimurium in complex samples, though it was not as sensitive as in culture, probably due to the complex ingredients in the milk. Meanwhile, we can see from Table 3 that our method was still slightly better than other methods in detecting *Salmonella* bacteria in actual milk samples. 

## 4. Discussion

Normally, magnetic capture efficiency mainly relies on antibody loading, but the antibody is large in size, and its activity is strongly influenced by the environment. In order to solve this problem, researchers chose a small–volume aptamer that can be loaded on the surface of magnetic beads and has high buffer tolerance. The final magnetic capture rate reached more than 90%, which was consistent with the results of Wu et al. [38] and was higher than that of Shan et al. [39], who selected antibodies. However, researchers spend a slightly longer time in this process, which could be related to the usage of micron-sized magnetic beads. Compared with nanometer-sized magnetic beads, their Brownian motion is slower and the reaction speed is slightly lower [40].

In this experiment, the salt-aging method was used to prepare AuNPs probes, and the gradually increasing concentration of NaCl in the buffer was used to counteract the electrostatic repulsion between ssDNA and citrate coated AuNPs, allowing a large number of sulfhydryl ssDNA can be assembled on the surface of AuNPs with high density until saturated. However, the amount of ssDNA used in the synthesis of the two probes is not the same, which could be related to the various conformations of distinct ssDNA sequences and their effects. 

To our knowledge, the difference in method sensitivity could be attributed to maker types. Thus far, the formation of numerous AuNPs by hybridization between ssDNA has rarely been observed to improve the sensitivity of the LFA. Similar studies, such as Pan et al. [9] and Zhong et al. [18], have used an antibody–antigen system to achieve gold aggregation. However, studies have shown that AuNPs with a diameter of 13 nm can bind to about 30 strands of ssDNA in 0.16 M NaCl, significantly increasing the ability of AuNPs probe to capture the target, which is unachievable with antibody [41]. The LOD of this research was better than those of Pan et al. and Zhong et al., confirming the principle. The results derived from Table 3 showed that the LOD enhanced by multiple AuNPs is comparable to that of fluorescent materials, and the results are intuitive [33,34]. At the same time, the method was controllable and nontoxic, and the materials were simple and easy to obtain. Although the detection limit of this study is lower than the Au^MBA^@Ag–RPA–SERS, it does not require complicated nucleic acid amplification. However, the Au^MBA^@Ag–RPA–SERS method has undergone three steps of complex material synthesis, RPA, and SERS enhancement, and therefore, primer mismatches are inevitable. Defects such as instability of composite materials and invisible results. Additionally, unlike rapid on-site testing, our research does not require pro–enrichment.

## 5. Conclusions

In order to realize the highly sensitive detection of *S.* Typhimurium in milk, the paper carried out the research of enhanced LFA. Through aptamer–magnetic separation, ssDNA1 signal transduction, and multiple AuNPs amplification by nucleic acid strand hybridization, the matrix interference was reduced; the complicated nucleic acid amplification was avoided; a large amount of AuNPs was gathered on the T-line and needed no pre-enrichment. Under optimized conditions, the LOD was 8.6 × 10^0^ CFU/mL in pure culture and 4.1 × 10^2^ CFU/mL in real samples by the naked eye, which was 1000 orders to the unenhanced LFA. In addition, the system exhibited prominent resistance to other pathogenic bacteria. This method was proven to be highly sensitive, controllable, simple, and time efficient, and it can be extended to the detection of other harmful substances, which will provide a broad application prospect for the detection of pathogenic bacteria.

## Figures and Tables

**Table 1:** The sequence of bio–aptamers, ssDNA was used in this study.

Name	Sequence (5′-3′)
Bio-aptamer	Biotin-TATGGCGGCGTCACCCGACGGGGACTTGACATTATGACAG
ssDNA1	TAACGTAGGAGGCATATATCAACATAATGTCAAGTCCCCGTCG
ssDNA2	SH-C6-TTTTTTTTTTTTTTTTTTTTTTTTT
ssDNA3	SH-C6-AAAAAAAAAAAAAAAAAAAAAAAAA
ssDNA4	SH-C6-TTGGGACTTGACATTATGACA
C-ssDNA	Biotin-AAAAAAAAAAAAAAAAAAAAAAAAA
T-ssDNA	TTGATATATGCCTCCTACGTTACCA-Biotin

**Table 2:** Specificity analysis of enhanced LFA for *Salmonella* Typhimurium.

No.	Strain	Source ^a^	Results
1	*Salmonella* Typhimurium	ATCC 14028	+
2	*Salmonella* Typhimurium	CMCC 50222	+
3	*Salmonella* Enteritidis	CICC 21482	−
4	*Salmonella* Dublin	CMCC 50042	−
5	*Salmonella* Dublin	CMCC 50092	−
6	*Escherichia coli* O157:H7	ATCC 25922	−
7	*Bacillus cereus*	CMCC 63303	−
8	*Enterobacter aerogenes*	ATCC 13048	−
9	*Cronobacter sakazakii*	ATCC 29544	−
10	*Listeria monocytogenes*	ATCC 19112	−
11	*Listeria welshimeri*	ATCC 43550	−
12	*Staphylococcus aureus*	ATCC 13565	−
13	*Shigella flexneri*	CMCC 51572	−
14	*Vibrio parahaemolyticus*	ATCC 17802	−

^a^ ATCC = American Type Culture Collection (Manassas, VA, USA); CMCC = China Medical Culture Collection (Beijing, China); + = positive results; − = negative results.

**Table 3:** Comparison with the different enhanced LFA detecting *Salmonella* bacteria in milk.

Method	LOD CFU/mL	Detection Range CFU/mL	Reference
Multifold AuNPs	4.1 × 10^2^	4.1 × 10^2^–4.1 × 10^7^	this work
AuNPs growth	10^4^	-	[32]
Upconverting phosphor	10^4^	10^4^–10^7^	[33]
Fluorescent–magnetic	3.75 × 10^3^	-	[34]
Gold-coated magnetic	10^3^	10^3^–10^6^	[35]
Silica-core quantum dot-shell	5 × 10^2^	10^2^–10^4^	[36]
DRS ^a^	10^2^	10^3^–10^7^	[11]
Au^MBA^@Ag-RPA-SERS ^b^	27	10^1^–10^6^	[37]

^a^ DRS: dual recognition strategy (ampicillin and antibody) and magnetic enrichment. ^b^ Au^MBA^@Ag–RPA–SERS: recombinase polymerase amplification–surface-enhanced Raman scattering.

## Data Availability

All the sequences of isolated strains and other raw data were submitted to the National Center for Biotechnology Information (NCBI). All the accession numbers have been shown in the materials and methods section.
